# Predicting the Effect of Mutations on Protein-Protein Binding Interactions through Structure-Based Interface Profiles

**DOI:** 10.1371/journal.pcbi.1004494

**Published:** 2015-10-27

**Authors:** Jeffrey R. Brender, Yang Zhang

**Affiliations:** 1 Department of Computational Medicine and Bioinformatics, University of Michigan, Ann Arbor, Michigan, United States of America; 2 Department of Biological Chemistry, University of Michigan, Ann Arbor, Michigan, United States of America; Iowa State University, UNITED STATES

## Abstract

The formation of protein-protein complexes is essential for proteins to perform their physiological functions in the cell. Mutations that prevent the proper formation of the correct complexes can have serious consequences for the associated cellular processes. Since experimental determination of protein-protein binding affinity remains difficult when performed on a large scale, computational methods for predicting the consequences of mutations on binding affinity are highly desirable. We show that a scoring function based on interface structure profiles collected from analogous protein-protein interactions in the PDB is a powerful predictor of protein binding affinity changes upon mutation. As a standalone feature, the differences between the interface profile score of the mutant and wild-type proteins has an accuracy equivalent to the best all-atom potentials, despite being two orders of magnitude faster once the profile has been constructed. Due to its unique sensitivity in collecting the evolutionary profiles of analogous binding interactions and the high speed of calculation, the interface profile score has additional advantages as a complementary feature to combine with physics-based potentials for improving the accuracy of composite scoring approaches. By incorporating the sequence-derived and residue-level coarse-grained potentials with the interface structure profile score, a composite model was constructed through the random forest training, which generates a Pearson correlation coefficient >0.8 between the predicted and observed binding free-energy changes upon mutation. This accuracy is comparable to, or outperforms in most cases, the current best methods, but does not require high-resolution full-atomic models of the mutant structures. The binding interface profiling approach should find useful application in human-disease mutation recognition and protein interface design studies.

## Author Summary

Few proteins carry out their tasks in isolation. Instead, proteins combine with each other in complicated ways that can be affected by either the natural genetic variation that occurs among people or by disease causing mutations such as those that occur in cancer or in genetic disorders. To understand how these mutations affect our health, it is necessary to understand how mutations can affect the strength of the interactions that bind proteins together. This is a difficult task to do in a laboratory on a large scale and scientists are increasingly turning to computational methods to predict these effects in advance. We show that by looking at the multiple alignments of similar protein-protein complex structures at the interface regions, new constraints based on the evolution of the three dimensional structures of proteins can be made to predict which mutations are compatible with two proteins interacting and which are not.

This is a *PLOS Computational Biology* Methods paper

## Introduction

The formation of protein-protein complexes plays an essential role in the regulation of various biological processes. Mutations play fundamental roles in evolution by introducing diversity into genomes that can either be selectively advantageous or cause a change in protein affinity that can result in malfunction of the protein interaction network [1, 2]. The Human Genome Project has yielded a wealth of data concerning natural human genetic variation that remains to be fully utilized. For example, it is well known that different people with the same condition often respond differently to the same treatment. A treatment that is effective in one population may have no effect or even be deleterious in another population. Knowledge of how individual subpopulations respond to drugs therefore remains a major bottleneck within the drug discovery process. Understanding how this natural variation within the human genome impacts the protein interaction network is expected to yield insight into this process, provided that the impact of a mutation on the formation of a protein complex can be reliably predicted. The rational design or modification of the affinity and specificity of protein-protein interactions is another challenging issue that has stimulated considerable efforts, as it presents many promising applications, notably for both industrial and therapeutic purposes [3].

Most of these efforts involve the prediction of the effect of a mutation upon the Gibbs free energy change of protein-protein binding (ΔΔG) on a large scale. Quantitatively, ΔΔG values for protein interactions may be measured experimentally by a variety of biophysical techniques [4, 5]. However, these methods are, with few exceptions, inherently low-throughput due to the need to express and purify each individual mutant protein before measurement. Alternatively, deep mutational scanning can be coupled with functional selection to analyze the effect of a large number of mutations on protein binding at specific sites within a protein [6, 7]. Deep sequencing is a very powerful method that has generated impressive insights into residue-specific contributions to protein binding. However, this method is still in its infancy and routine application is still difficult.

As a result, scientists have increasingly turned to computational methods to predict ΔΔG values. For a rigid protein, the ΔΔG of folding or protein binding can be determined relatively accurately from a full-atomic description of the protein structure or complex, using either potentials based on molecular mechanics that attempt to quantify the interactions in physically meaningful terms [8], statistical potentials based on the likelihood of similar interactions and local conformations occurring in the PDB [9], or some combination of the two. However, this approach ignores the structural changes that can occur upon mutation, which can alleviate clashes and position residues in conformations more favorable for interaction. Accordingly, much research has gone into the incorporation of flexibility into energetic calculations [8, 10–12]. However, the method is computationally expensive for large datasets to the extent that it becomes prohibitive for genome-wide studies or even scanning mutations on a single protein. In addition, in many cases, a more exact physical representation of the molecular structure and interactions have proved to be less accurate than simpler models due to the inherent inaccuracy of each term in the force-field.

As such, alternative methods have been proposed that either use reduced representations of the protein structure or simplified interaction schemes (for example, the use of Cβ and contact potentials) [13, 14] or omit the atomic details of the structure entirely and use machine-learning to predict ΔΔG values from sequence conservation or from gross structural features such as solvent accessibility and secondary structure. The accuracy of a machine learning method ultimately depends on the quality of the feature set and the experimental data available to train the method. If the training set is representative, completely covering all relevant types of interactions and not significantly biased towards specific interactions, it is possible to use machine learning to accurately predict the effect of a mutation using features that are only weakly predictive on their own. If the training set is not representative, then a model formed from only weak predictors is usually not generalizable [15]. The effect of mutations on protein stability has been heavily studied experimentally and non-redundant datasets have been constructed that are believed to be representative of all classes of possible interactions. By contrast, information on the effects of mutations on protein complex formation is much more limited with the data heavily focused on only a few interaction types [16]. For this reason, constructing a machine learning method for the prediction of ΔΔG values for protein complex formation is more difficult than constructing a machine learning method for stability predictions [9]. As a result, the resulting methods generally have a lower accuracy compared to protein stability predictions [9]. Furthermore, the models are usually less generalizable and often show large drops in accuracy when tested on new data not in the training set.

This limitation can be overcome if new and more accurate predictors are available for ΔΔG prediction. Because physics based features often share the same limitations, attempts have been made to predict ΔΔG using alternate scoring methods. Based on their success in the prediction of ΔΔG values for protein stability [17, 18], sequence based features have been suggested as predictors of protein-protein interaction ΔΔG values [19]. Protein binding affinity is under evolutionary pressure and we expect residues that contribute strongly to binding energetics to be more strongly conserved than residues which have minimal impact on binding. The conservation of binding residues plays an important role in many “hot spot” prediction programs which seek to identify sites on the interface which strongly influence binding [20]. Taking this approach further, it is likely that the observed distribution of amino acids at a site within the interface reflects at some level the amino acid energetic preferences for binding. Other things being equal, the probability of finding an amino acid which unfavorably impacts binding at an interface site will be less than finding a more favorable amino acid—provided that affinity, and not some other property, is the driving force for selection.

However, in many cases there are other driving forces for selection besides protein-protein binding affinity such as binding specificity [6, 21, 22], foldability [23], or protection against aggregation [24]. In addition, closely related sequences bear the imprint of their evolutionary relationship independent of any functional relationship [25]. The limited time of divergence from a common evolutionary ancestor creates a phylogenetic signal that can complicate analysis as not all possible mutations are effectively sampled during the divergence time [26]. Both effects can be reduced by considering structurally similar interfaces rather than closely evolutionarily related proteins. Structurally similar interfaces are expected to serve similar roles regardless of their evolutionary relationship; an effect that can be seen by the existence of highly similar interfaces in proteins that are otherwise structurally dissimilar and evolutionarily distant [27].

Using this approach, we show an interface binding profile score, called BindProf, formed from an aligned ensemble of structurally similar interfaces has accuracy as a standalone feature similar to, or in most cases, better than many composite all-atom potentials. Unlike the all-atom energies, it can be calculated very rapidly once the profile is constructed. The on-line server of the BindProf program is freely available at http://zhanglab.ccmb.med.umich.edu/BindProf.

## Results

**Fig 1:**
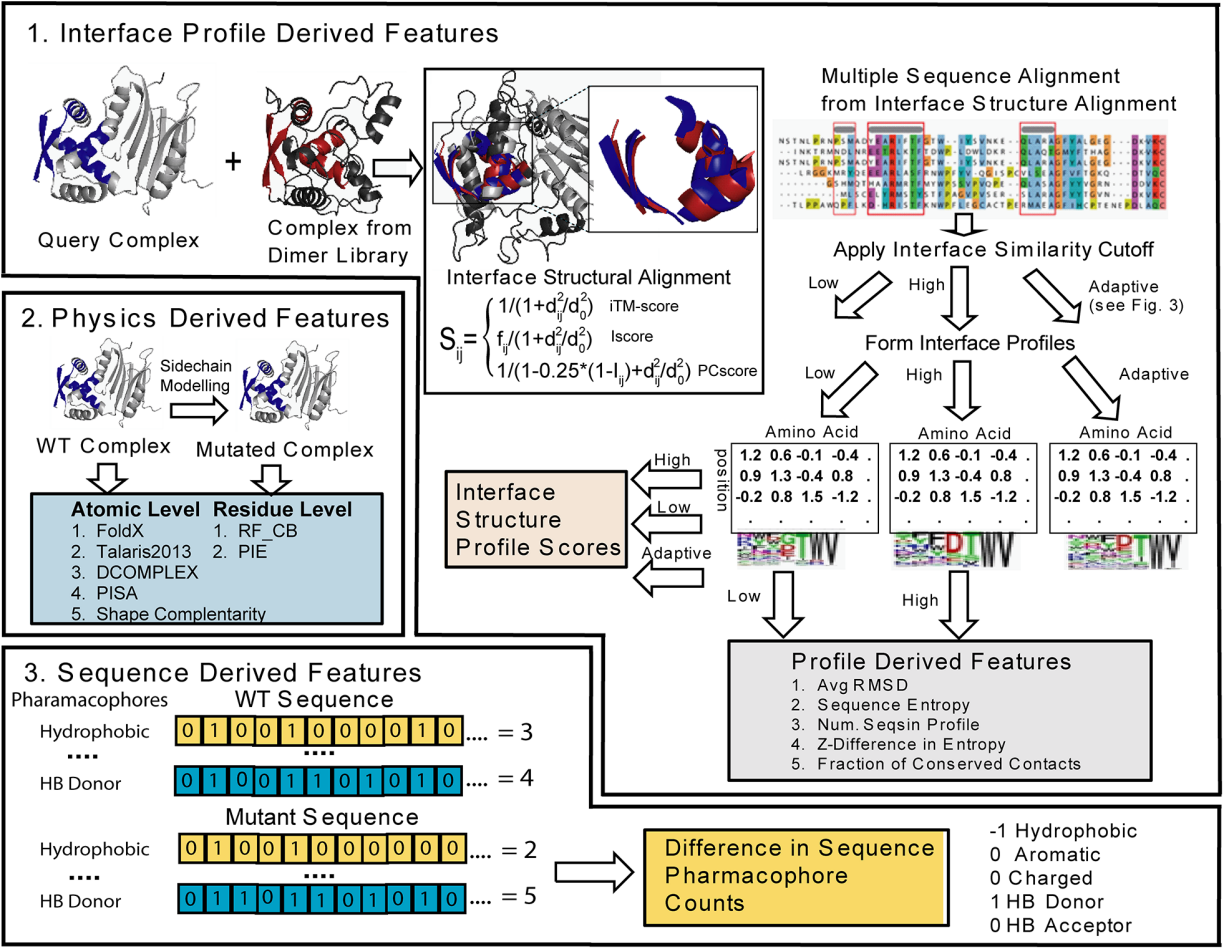
Pipeline of BindProf for predicting protein-binding affinity using features derived from interface structural profiles, wild type (WT) and mutant sequences, and physics based scoring of the structures of the WT and mutant complexes. (**1**) Interface profile scores and Interface profile scores features are derived by profile scoring structural alignment of structurally similar interface using an interface similarity cutoff to define the aligned sequences that are used to build the profile. (**2**) Physics based scores are formed at the residue or atomic level formed by modeling the mutant monomeric protein and complex and evaluating the difference in energy. (**3**) Sequence features are formed by the difference between the WT and mutant sequences in the number of hydrophobic (V, I, L, M, F, W, or C), aromatic (Y, F, or W), charged (R, K, D, or E), hydrogen bond acceptors (D, E, N, H, Q, S, T, or Y), and hydrogen bond donating residues (R, K, W, N, Q, H, S, T, or Y) along with the difference in amino acid volume calculated from the sequence.

To predict the free-energy change of protein-protein interactions, ΔΔG, BindProf adopts a multi-scale approach shown in Fig 1 using a variety of features at different levels of structural resolution using machine learning with sequence and structure based features to learn the correct weighting between terms using a regression tree classifier. A unique feature of BindProf is the inclusion of a structural profile score reflecting the likelihood of a given sequence being found in the ensemble of structurally similar protein-protein complexes. Since function follows structure more closely than sequence, we expect the structural profile score to more accurately reflect ΔΔG changes than sequence conservation. Such an expectation has been borne out in our protein design program EvoDesign [28, 29], where the structural profile score was found to be the dominant factor in a multi-scale approach that resulted in the majority of tested sequences experimentally folding to the designated structures.

### Mutant Interface Profile Scores As a Predictor of ΔΔG for Complex Formation

Since the most distinctive feature of our approach is the use of structurally similar interfaces of protein complexes in the PDB to score the effect of a mutation, we first consider the most accurate way to predict ΔΔG of protein binding using only this information. The amino acid distribution of structurally similar complexes can be analyzed quantitatively by the use of structural profile scores. Similar to a position specific scoring matrix, a structural profile score *F*(*p*, *a*) reflects the log odds likelihood of an amino acid (*A*) being found at a particular position (*p*) in an aligned ensemble of structurally similar proteins [30]

$$F(p,A)=\sum_{a=1}^{20}g(p,a)M(A,a)$$ (1)

where *g*(*p*, *a*) is the Henikoff weighted frequency of the amino acid *a* appearing at the *p*th position in a multiple sequence alignment (MSA) with exactly redundant interface sequences removed; *M*(*A*, *a*) is the BLOSUM substitution matrix with *a* varying for 20 amino acids, which is used to account for missing structures in the PDB. Experimental ΔΔG values are therefore hypothesized to be proportional to the mutant profile score defined as the difference between the profile scores of the wild type (WT) and mutant (Mut) amino acids at position *p* in the interface:

$$\Delta \Delta G_{\text{calc}}=\sum_{a=1}^{20}g(p,a)M(A_{\text{WT}},a)-\sum_{a=1}^{20}g(p,a)M(A_{\text{Mut}},a)$$ (2)

The profile therefore depends on both the cutoff level for defining a similar complex and the measure of similarity used. The definition of “similar” is less straightforward in regards to interfaces than it is with overall protein structure. Similarity of protein structures can be defined by a normalized, length independent measure of structural difference, TM-score, which has been shown to have a close relationship with fold classification [31, 32]. For interfaces, a straightforward definition is to use the normal procedure for the structural comparison of proteins but to only consider interface residues in the comparison [33]. A similar interface in this case is defined as having a high TM-score when only residues at a given cutoff distance (4 Å) from the other chain are considered for alignment and scoring (iTM-score, see definition in Methods) [33]. A more stringent comparison (Iscore) can be made by considering not only backbone alignment but also contact patterns at the interface to more clearly distinguish closely related proteins [33]. Finally, even close structural matches can result in significantly different binding energetics if there is a mismatch of interaction types at the interface. For example, the mutation of hydrophobic to a charged residue can result in a severe loss of affinity if the mutation is located within a hydrophobic pocket. Accordingly, the alignment can be modified to take into account physicochemical similarity during alignment using a pharmacophore classification of residues to identify residue similarity (PCscore) [34].

**Fig 2:**
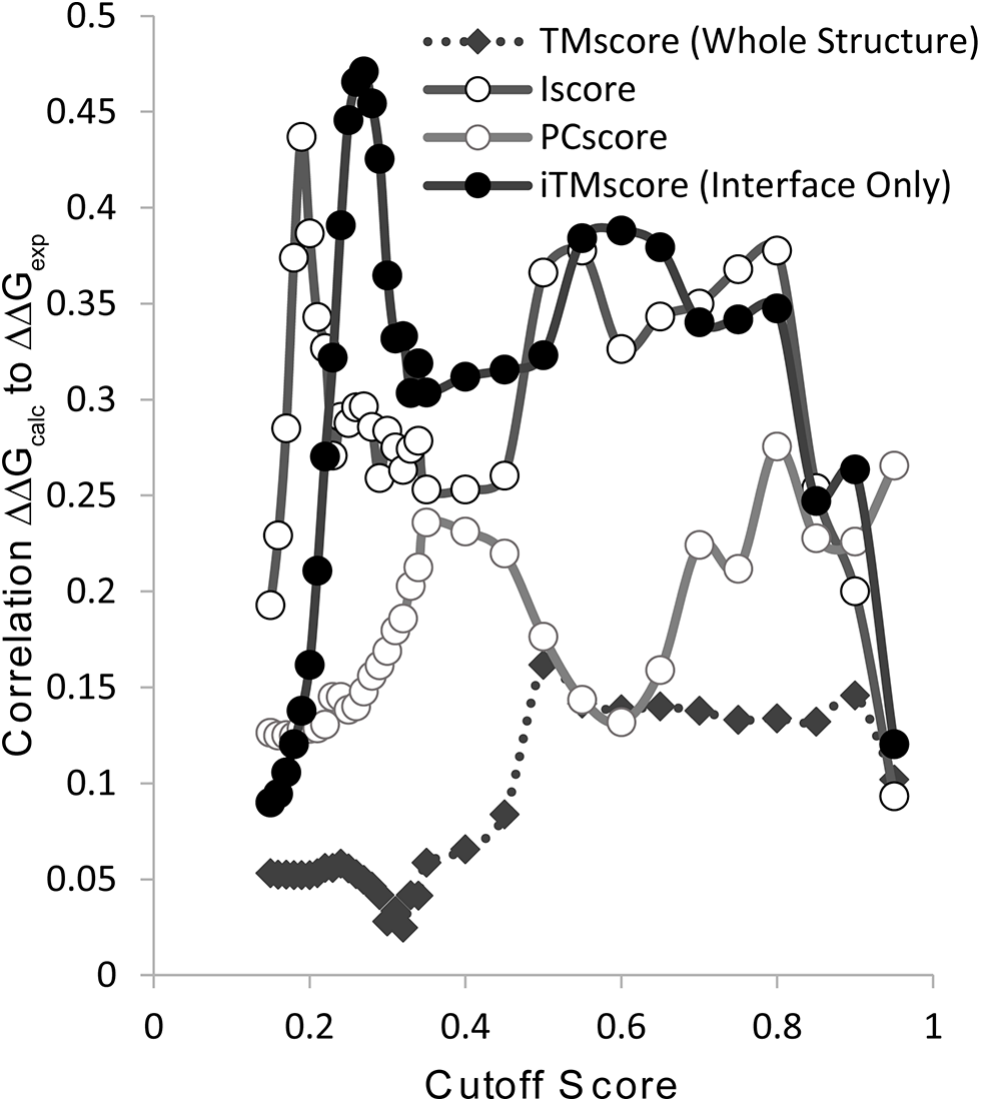
Comparison of the accuracy of mutant interface profile scores formed from different structural alignment methods in predicting ΔΔG of complex formation. The iTM-score considers only structural similarity at the interface, Iscore considers structural similarity at the interface and the fraction of native contacts preserved, and PCscore considers both physicochemical and structural similarity at the interface. TM-score considers only structural alignment of the mutated monomeric protein. Profiles are constructed from sequences meeting each cutoff and the predicted ΔΔG values are calculated according to Eq 2.

In Fig 2 we show the correlation between ΔΔG values calculated by the mutant interface profile scores (Eq 2) and experimental ΔΔG values of protein-protein interactions from the SKEMPI database [16] as a function of the alignment methods and cutoff values. Each method shows the expected general rise and fall in the accuracy at extreme values as the cutoff is made either too strict or too loose. Too loose cutoffs degrade the accuracy of the profile score as structurally unrelated complexes are included in the profile and the specific information from structurally related complexes is lost. Too strict cutoffs, on the other hand, include too few sequences to construct an accurate profile that reflects all the actual allowable possibilities at the interface. While all similarity measures show low accuracy asymptotically at very high and low cutoff values, a simple unimodal distribution of accuracy with the cutoff value is only observed for the profile score formed from structural alignment of the monomeric protein. In this case, the accuracy of the profile score reflects the underlying bimodal distribution of the TM-Score, which has a sharp division near TM-Score cutoff values of 0.5 separating similar folds from unrelated structures [32]. Since TM-Scores of 0.5 and above correspond with high probability to similar folds while a TM-Score below this value indicates essentially no relationship between structures [32], the monomeric profile score is only accurate above a TM-Score 0.5. However, the actual correlation with the experimental ΔΔG values is modest and the profile scores from all interface alignment methods yield a significantly better correlation for nearly the entire range of cutoff values.

The relationship between cutoff value and ΔΔG prediction for the interface alignment methods (iTM-score, Iscore, and PCscore) is more complex reflecting a more complex underlying distribution. In each case, the accuracy of ΔΔG prediction is at least bimodal with the cutoff value. Like the monomeric structure profile, the accuracy rises at strict cutoff values. As the cutoff is reduced it levels off as an adequate representation of closely related complexes is built. However, unlike the monomeric structure profile, the accuracy rises again at lower cutoff values, eventually reaching a higher accuracy than can be achieved by profiles constructed from closely related complexes. Closer inspection of the actual origin of this effect is the inclusion of sequences at lower cutoff values that can be aligned accurately to a region within the interface but with relatively poor overall global alignment. From the viewpoint of applications which rely on global properties like the recognition of convergently evolved similar interfaces for function annotation [35–38], these sequences are less useful as they reflect similarity in only a small region of the interface. However, on a physical level, binding interactions are fundamentally local properties. In the interior of a protein, amino acids are tightly packed and a mutation at one site can cause a rearrangement of the protein core [39]. At the interface, however, packing is less tight and a considerable fraction is exposed to solvent even in the protein complex [40]. The difference in packing gives a conformational freedom at the interface that is not present in the interior which can retard the propagation of packing defects throughout the interface after a mutation [41]. With this in mind, the relative inaccuracy of profiles based on PCscore alignment at predicting ΔΔG values can be explained, despite the fact that PCscore is the only method that attempts to incorporate physicochemical similarity into the alignment procedure. Because PCscore penalizes amino acid mismatches more severely, more sequences with good local matches but poor global similarity are missed.

**Fig 3:**
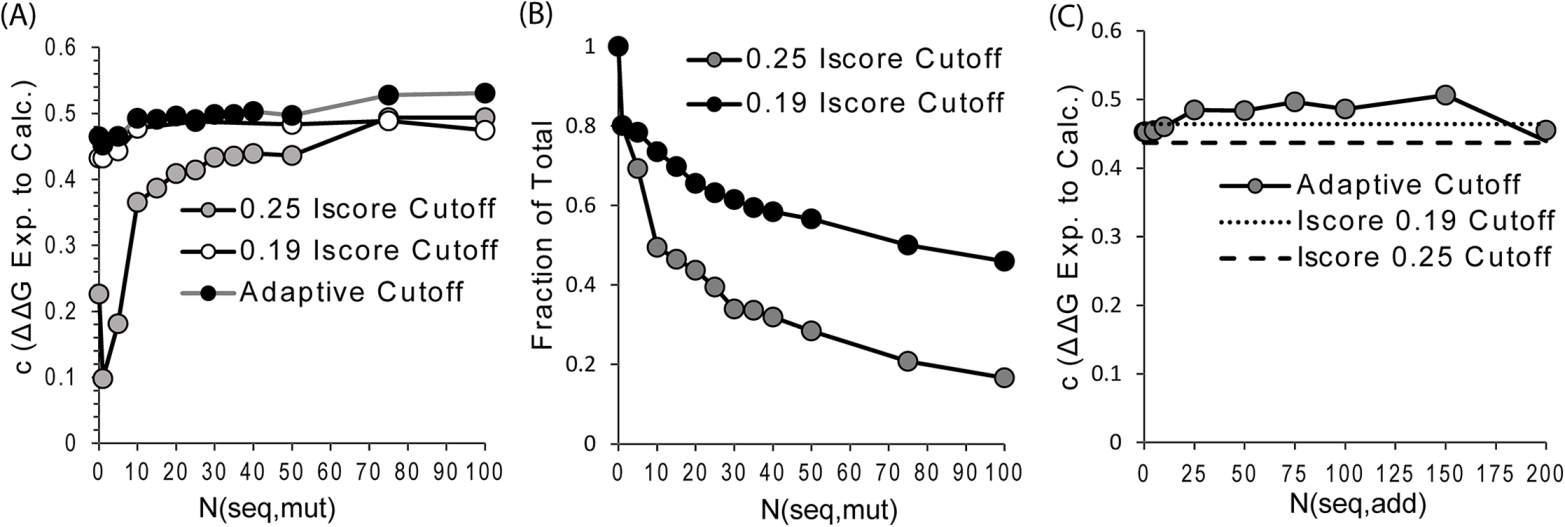
Dependency of the accuracy of ΔΔG prediction on the number of sequences that can be aligned at the site of the mutation and the formation of an adaptive profile mixing sequences from high and low interface similarities. Only single site mutations are considered (81% of the total number of mutations). *N*_*seq*,*mut*_ and *N*_*seq*, *add*_ are the number of sequences that can be aligned at the site of the mutation and the number of lower similarity sequences added to the profile, respectively. **(A)** Pearson’s correlation *c* between predicted and experimental ΔΔG values as a function of the number of sequences that can be aligned at the site of the mutation. **(B)** Fraction of the total number of single site mutations as a function of the number of sequences that can be aligned at the site of the mutation. **(C)** Improvement in accuracy of an adaptive profile mixing sequences from high and low interface similarities over profiles formed purely using high and low interface similarity cutoffs.

Taken individually, sequences with higher interface similarity should be more predictive of ΔΔG then sequences with lower interface similarity. However, the accuracy of the interface profile score is highly dependent on the number of sequences that can be aligned at the site of the mutation. A representative example is shown in Fig 3A. At a high interface similarity cutoff (IScore = 0.25), the accuracy of the profile score rises steeply until about 15 sequences can be aligned at the position, mirroring a similar result for protein stability [28, 29]. At low interface similarity (IScore = 0.2), the number of sequences is less predictive of the accuracy of the profile score, likely because a sufficient number of sequences can be found for all positions except those at the extreme edge of the interface (see below).

We therefore considered an adaptive procedure to form a more accurate profile. The sequences are first sorted by descending interface similarity. All sequences with an interface similarity above a strict cutoff are added to the profile and up to *n* sequences are added until the second, looser cutoff is reached. Fig 3C shows the improvement in ΔΔG prediction from Iscore alignment as a function of *n* for the optimal high and low interface similarity cutoff values (IScore = 0.25 and 0.19). *n* reaches a shallow maximum around 80 sequences. The adaptive profile shows a significant improvement over the profile formed from a high similarity cutoff and a smaller improvement over the profile formed from a high similarity cutoff.

### The Interface Profile Score Has Accuracy Superior or Comparable to Other Potentials

To assess the potential of interface profile scores for either standalone ΔΔG prediction or as a feature in machine learning based score combinations, we compared the accuracy of interface profile scores formed from high, low, and adaptive profiles by Iscore alignment to a diverse set of multi-scale potential terms. Although iTM-score profiles are slightly more accurate than Iscore profiles at predicting ΔΔG (Fig 1), we chose Iscore profiles for comparison because an additional feature calculated from the profile, the fraction of conserved contacts, can be used to predict the accuracy of the profile score for machine learning. The tested set of potentials includes: the all-atom empirical potential FoldX [42, 43], a composite statistical and physics based potential from Rosetta (Talaris 2013) [44], residue and all-atom docking potentials (PIE [45] and PISA [46], respectively), all atom and C_β_ based statistical potentials (DCOMPLEX [47] and RF_CB [48], respectively), a shape complementarity score [49], changes in the total, polar, and hydrophobic solvent accessible surface area (SASA), the difference in hydrogen bond counts across the interface in the structures of the WT and mutant complexes, the volume difference between WT and mutant residues, and pharmacophore count differences of hydrophobic, and aromatic and hydrogen bonding forming residues between the WT and mutant complexes [50].

**Fig 4:**
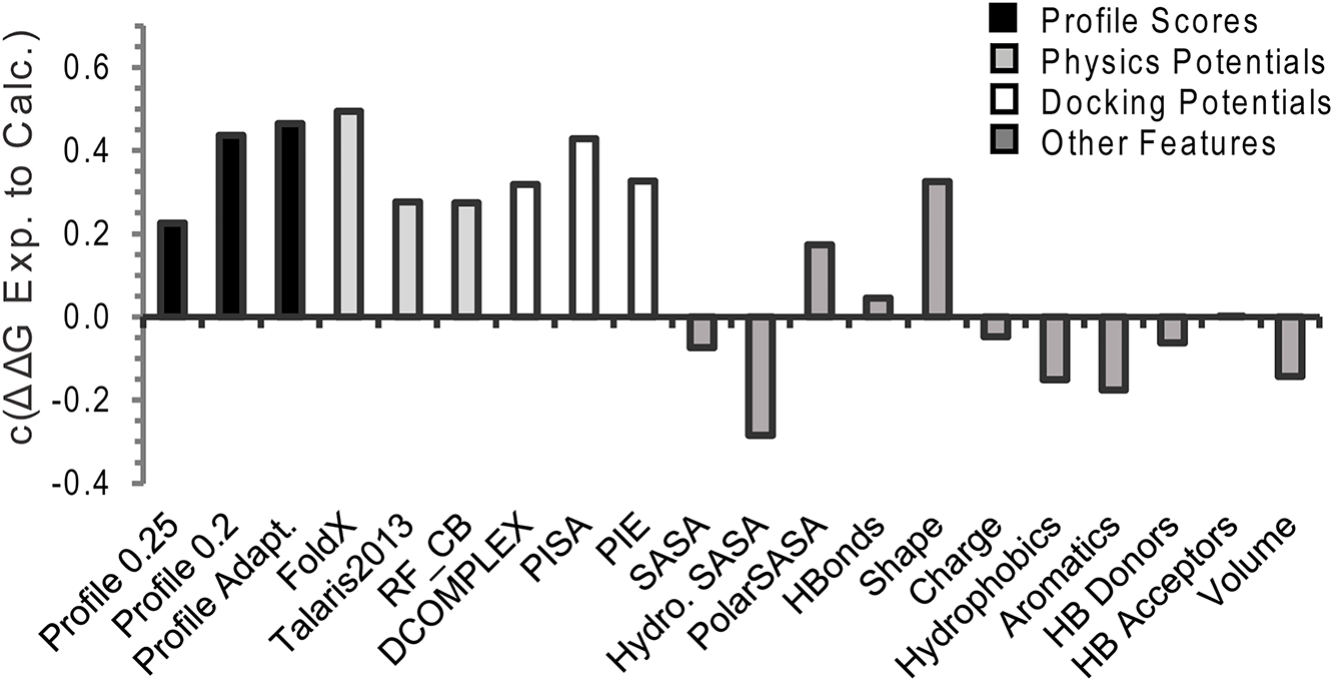
Comparison of the accuracy interface profile scores at ΔΔG compared to other physical, statistical, and sequence based potentials for all mutations in the SKEMPI dataset. See text for a description of each potential.

The Pearson’s correlation coefficient *c* between predicted and experimental ΔΔG values is shown in Fig 4 for the adaptive interface profile score and the multi-scale potentials described above. When all mutations are considered, the adaptive interface profile score is more accurate at predicting ΔΔG than all the other potentials considered except for FoldX. However, the difference in *c* between FoldX and the adaptive interface profile score is not statistically significant when using a two-tailed Fischer r-to-z transformation (p-value = 0.32). The difference in *c* between the adaptive interface profile score and the all-atomic docking potential PISA is also statistically insignificant (p-value = 0.2). The adaptive interface profile score is superior in accuracy to all other potentials tested at high statistical significance (p-value<0.001).

From Fig 4, FoldX appears the most accurate single method in terms of Pearson correlation coefficient *c* although it is statistically indistinguishable with BindProf and PISA. However, this value could be biased somewhat by the fact that the side-chains of the mutant have been reconstructed using the FoldX force field. A mismatch between the force field used to optimize the side-chain rotamers and the scoring potential can result in a degradation of the performance. In our early trials, the Talaris2013 Rosetta force field generally showed similar performance to FoldX values if the side-chains were reconstructed using the Talaris2013 forcefield.

We note that although the BindProf score compares favorably with other individual potentials, the Pearson correlation coefficient *c* is still relatively low (below 0.5). However, one of the key features of BindProf is that it works on a fundamentally different basis then the other methods that are currently in use. This complementarity should be of important help for improving the overall recognition accuracy of multiscale potentials when combined with other sources of potentials as demonstrated below.

### Interface Profiles Excel at Finding Favorable Mutations Compared to Other Methods

In many applications it is desirable to know the accuracy of ΔΔG prediction across different categories of experimental ΔΔG values. For example, the accuracy of predicting destabilizing mutations is significantly less important in protein design than the accuracy of predicting favorable mutations, as strongly destabilizing mutants are rejected during the design process. Any inaccuracy in prediction therefore only matters to the extent they are misclassified as favorable or neutral mutations. On the other hand, favorable mutations should be enriched during the design process and accurate ΔΔG prediction is essential for these mutations. We therefore recalculated the Pearson’s correlation coefficient *c* between experimental and calculated ΔΔG values restricting the dataset to the entries with experimental ΔΔG values within the appropriate range.

**Fig 5:**
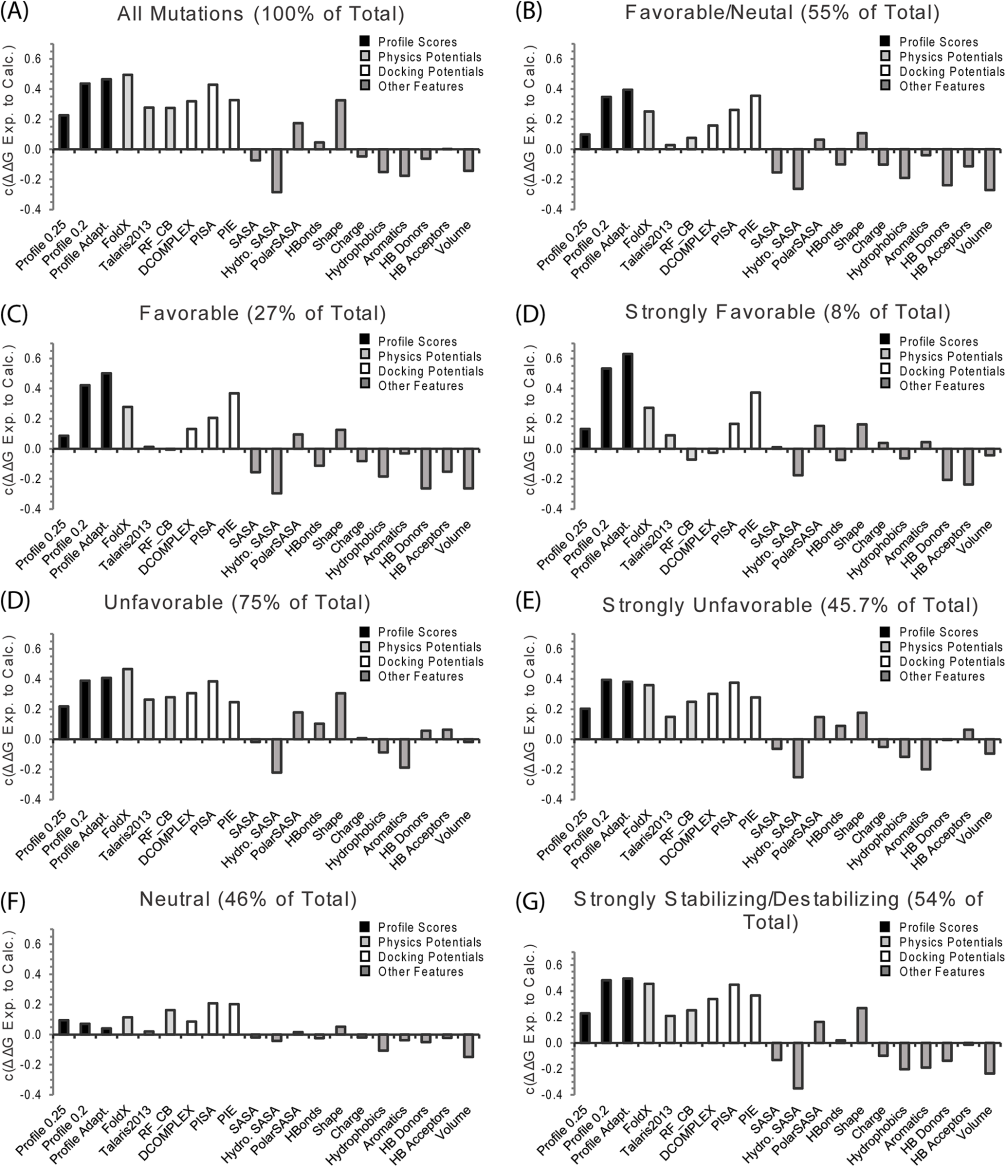
Breakdown of the performance of the interface profile score compared to other potentials for different classes of mutations. Favorable: ΔΔG ≤ 0 kcal/mol, Strongly Favorable ≤ -1 kcal/mol, Unfavorable: ΔΔG ≥ 0 kcal/mol, Strongly Unfavorable: ΔΔG ≥ 0 kcal/mol, Neutral ΔΔG ≤ 1 kcal/mol and ≥ 1 kcal/mol. See text for a description of each potential.

Interface profile scores show exceptional performance relative to other predictors (*c* = 0.5) at predicting favorable mutations (ΔΔG values ≤0 kcal/mol, 27% of the total, see Fig 5B). This is an important result as finding favorable mutations is a very important target for many applications, such as protein design to build more tightly binding interfaces, which have so far proven difficult to predict by physics based methods [12, 51]. The most predictive feature in most categories, FoldX, performs poorly here (*c* = 0.28 compared to *c* = 0.46 for destabilizing mutations), similar to previous observations which also included a degree of backbone flexibility by incorporating a short relaxation before the calculation of FoldX energies [51]. Likewise, other features like shape complementarity and the statistical potentials DCOMPLEX and RF_CB that normally perform well also perform poorly in this category. This effect is even more magnified when only strongly favorable mutations (ΔΔG values ≤ -1 kcal/mol, 8% of the total) are considered (Fig 5D).

Interface profiles are less accurate in predictions of unfavorable mutations (ΔΔG values ≥0 kcal/mol, 75% of the total in Fig 5E), likely because the statistics of unfavorable mutations are based on a lower number of frequency counts within the profile [17]. Full atomic physical potentials (FoldX and the Rosetta’s Talaris2013 score function) and docking potentials (PISA and PIE) do well in this category. Shape complementarity is also predictive of unfavorable mutations (*c* = 0.31) while it is not predictive of favorable mutations (*c* = -0.13). All methods were inaccurate in determining the subtle differences between neutral mutations (ΔΔG values between 1 and -1 kcal/mol, 46% of the total, Fig 5G). Fortunately, inaccuracies within this range are usually of less consequence since a mutation with a ΔΔG value between 1 and -1 is often tolerated with little impact on a protein’s function. However, the cumulative impact can be significant when multiple mutations are considered such as in protein design applications. Since all the methods are inaccurate within this range and only a small fraction of mutations are actually favorable, reverting mutations with predicted ΔΔG values >1 kcal/mol back to WT may be a successful strategy for loss of affinity in design proteins through the accumulation of many small errors.

### The Accuracy of Interfacial Profile Scoring for ΔΔG Prediction Can Be Inferred Based on the Changes in Relative Solvent Accessible Area upon Complex Formation

**Fig 6:**
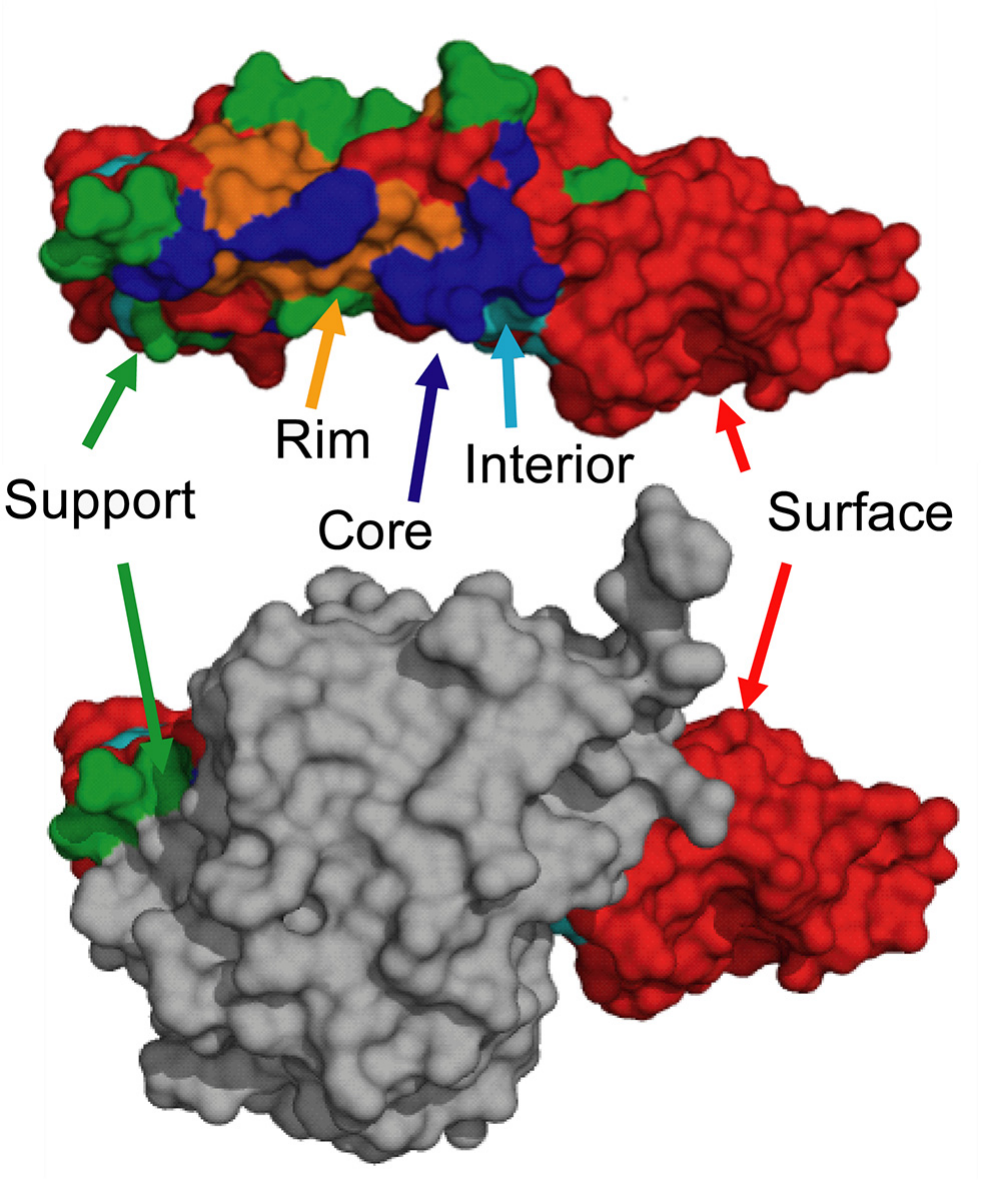
An illustration of the interface residue types onto the surface shown from the growth hormone-receptor complex structure (PDB ID: 1A22). The monomer structure of one of the chains is shown on top with the complex structure on bottom. ‘Core’ residues (blue) are exposed in the monomeric structure but buried in the complex; ‘Support’ residues (green) are partly buried in the monomeric structure and fully buried in the complex; ‘Rim’ residues (orange) are fully exposed in the monomeric structure and partly buried in the complex; ‘Interior’ residues (sky blue) are fully buried in the monomer, while surface residues (red) are fully exposed in both the monomeric and complex structures.

**Fig 7:**
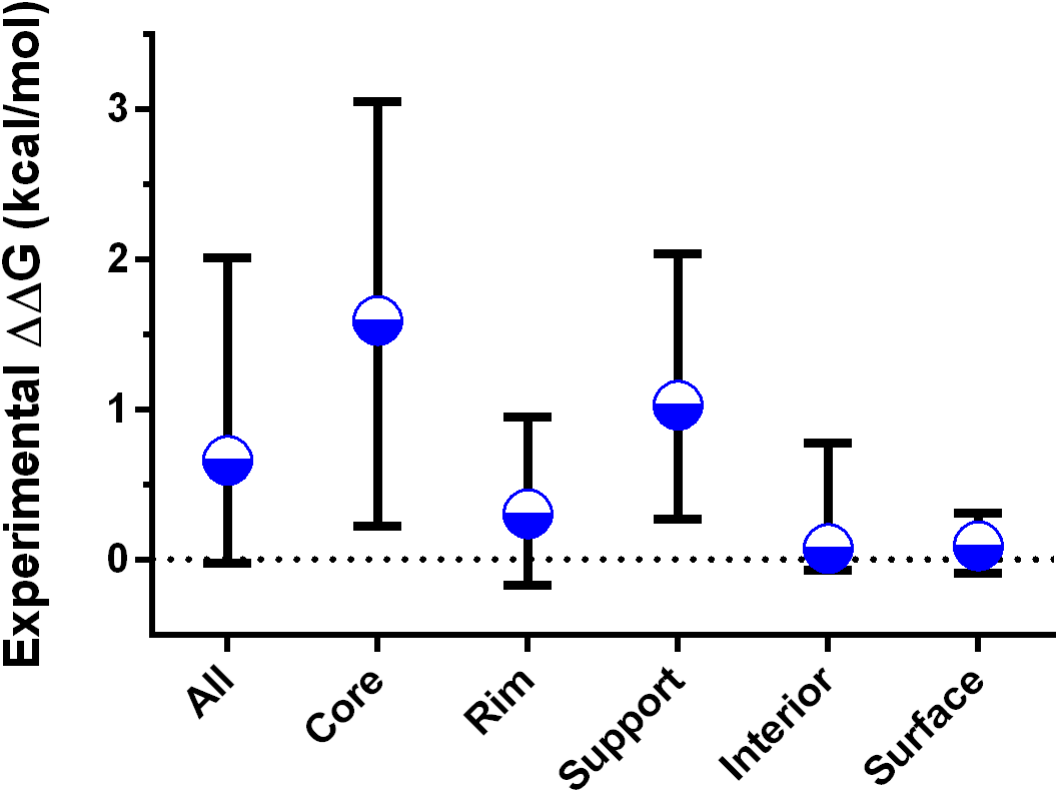
Median and interquartile ranges of experimental ΔΔG values by interface classification. Full distributions can be found in the Supporting Information as S1 Fig.

We next sought to see if the accuracy of interface profile scores could be predicted from the characteristics of the profile. Interface residues play different roles in protein-protein interactions and display both different conservation patterns and different types of interactions depending on their relative position within the interface [40]. Since the accuracy of both the interface profile scores and the sequence and physics based scores are expected to be sensitive to these changes, it is of interest to compare the accuracy of different methods based on the different types of interface residues. This requires that a standard classification of the roles that different residues play in binding be made, which is difficult if only their geometric position within the interface is considered. Instead, one of the most natural classification of interface residues for binding energetics is determined by comparing the relative solvent accessible area of the residue in the monomeric protein (rASA) to the relative solvent accessible area in the protein complex (rASA_c_) (Fig 6). Following Levy [40], the “core” residues are defined as residues which are exposed in the monomeric protein (rASA>25%) but buried in the protein complex (rASA_c_ <25%). Core residues are typically hydrophobic with a composition strongly divergent from the composition of the remainder of the protein surface [52]. Core residues supply the bulk of the energy driving association by hydrophobic interactions [53]. The hydrophobic interactions within the complex cause the core region to become tightly packed upon complex association with little room for conformational variability. For these reasons, the core residues are strongly conserved during evolution [53, 54], and mutations in this region are usually more strongly unfavorable when compared to mutations at the periphery of the interface (see Figs 7 and S1).

“Rim” residues surround the core residues and are also exposed in the monomeric protein. But unlike the core residues, the rim residues become only partially (0–25% rASA_c_) buried upon complex formation. The rim residues have a composition more similar to the surface of the protein away from the interface [52]. Rim residues are frequently charged and often engage in hydrogen bonding or salt bridges with the binding partner [53]. The rim residues help to alleviate protein aggregation by charge repulsion and can contribute to binding specificity by forming specific polar contacts with the binding partner. In some cases, the rim residues also tune the strength of binding, stopping the formation of an excessively stable complex which prevents the formation of other complexes within the interaction network. Most of the favorable mutations are found within this region, with the most common favorable mutation being a charge reversal which alleviates an unfavorable electrostatic interaction within the complex. Rim residues show much less sequence conservation than the core residues. Because of their role in the fine tuning of protein interactions and because the rim of the interface is less tightly packed [41] than the core residues, these residues are much less evolutionarily conserved.

“Support” residues are partially buried in the monomeric protein, and fully buried in the complex. As such, they are usually hydrophobic and located in the center of the interface near the core residues. However, because the change in surface area upon complex formation for support residues is less than core residues they are less important energetically and are subject to more sequence variation than the core residues.

The final two categories of “surface” (rASA_c_ >25% and rASA <25%) and “interior” (rASA_c_ <25% and rASA <25%) consist of residues that make no contacts with the binding partner. Mutations within these regions only influence complex formation indirectly by influencing conformational changes, by destabilizing protein folding [23, 55], or by long-range electrostatic interactions and alteration of the hydrogen-bonding network [56]. Consequently, they generally have a minimal impact on the energetics of complex formation (Fig 7).

**Fig 8:**
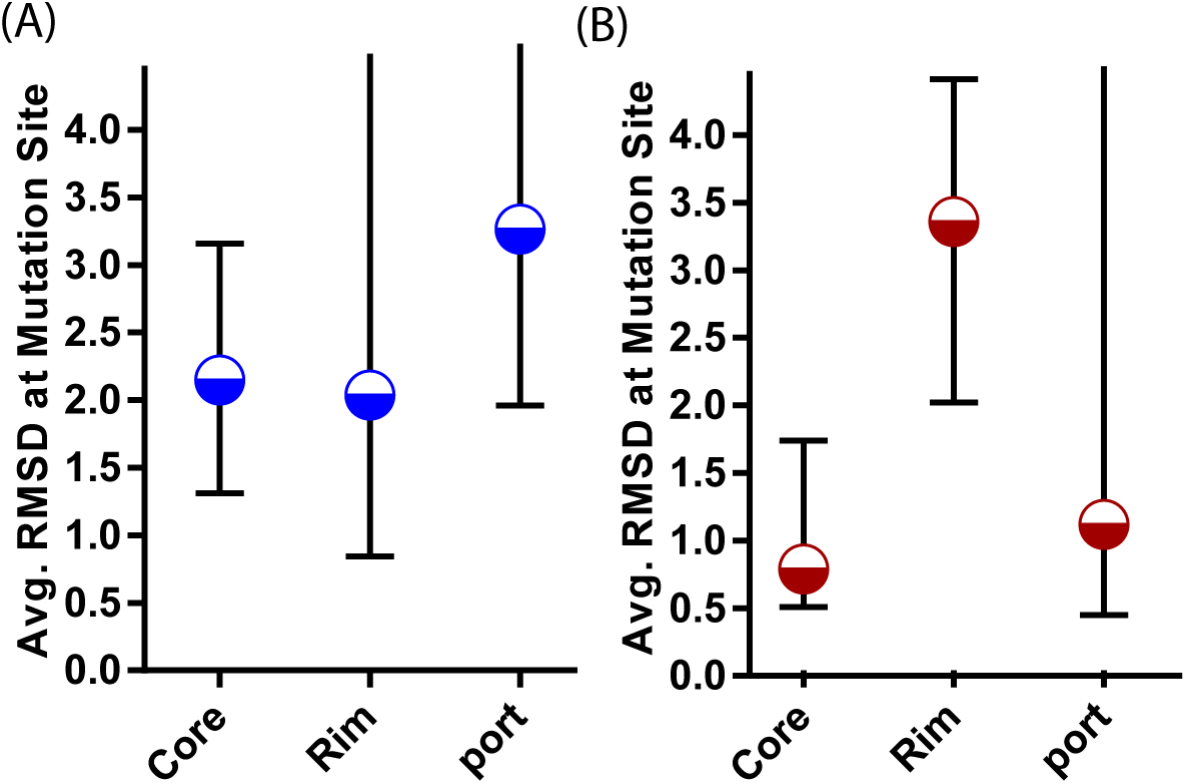
Median and interquartile ranges of the RMSD of the alignment at the mutation site at low (Iscore = 0.19) (A) and high (Iscore = 0.25) (B) interface similarity.

**Fig 9:**
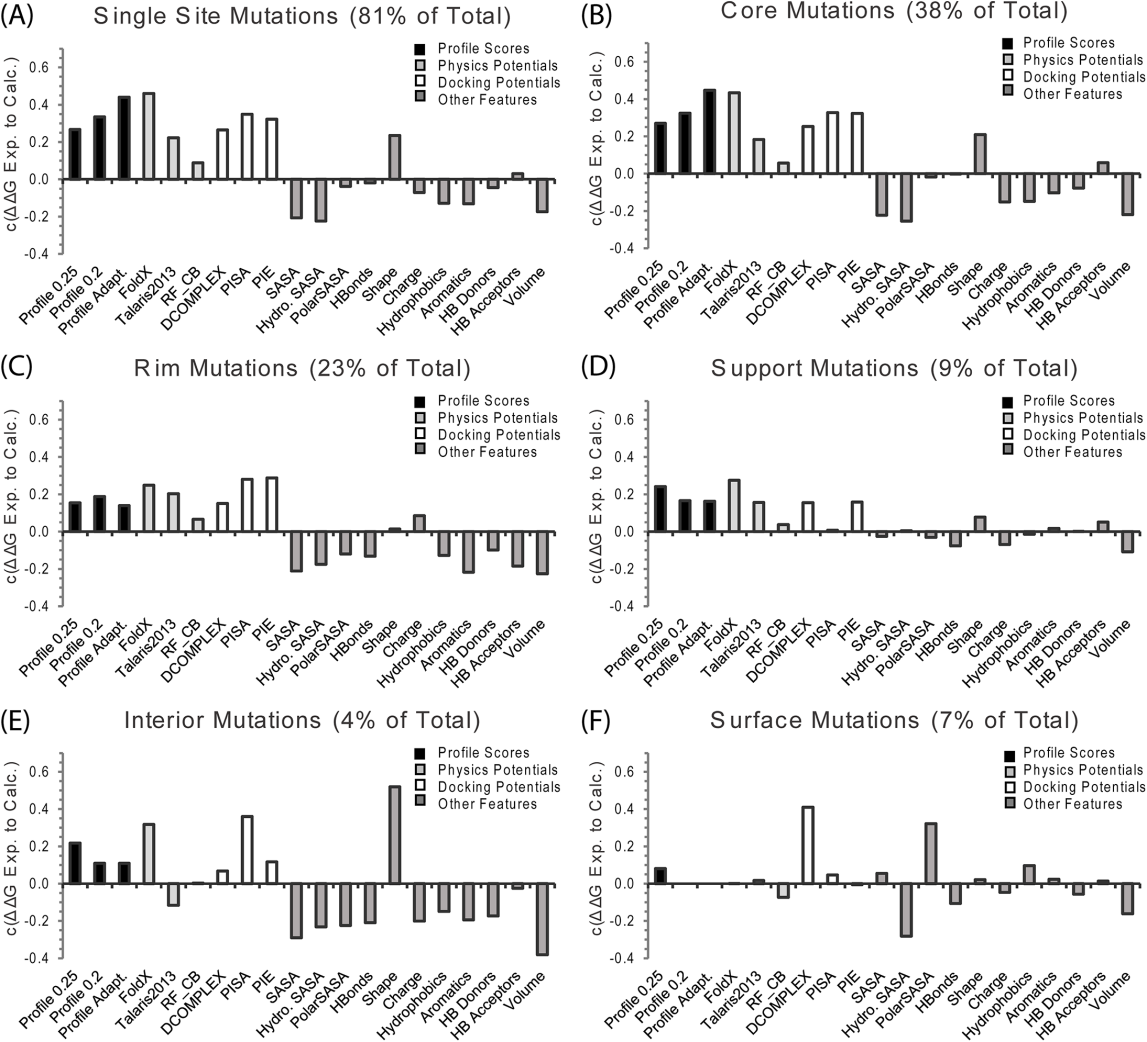
Breakdown of the performance of the interface profile score compared to other potentials for different types of interface residues. See Fig 6 for the definition of the interface residue types.

Since this classification by changes in rASA upon complex formation also indirectly reports on the position of the mutation within the interface, it is expected that the performance of the interface profile score will vary as well. The interface profile score is most accurate for the core residues (Fig 9) which are generally located at the center of the interface (Fig 6). The alignment is significantly more accurate in this region compared to the rest of the interface, especially when the cutoff is restricted to only highly similar complexes (Fig 8). The relative advantage of the interface profile score over methods is decreased when non-core residues are considered. The all-atom physics based potentials Talaris2013 and FoldX were also less accurate in predicting the ΔΔG of mutations outside the core residues, most likely because electrostatic and hydrogen bonding interactions are significantly more difficult to predict by physics-based methods than interactions primarily based on hydrophobic contacts [57]. Instead, the docking potentials PIE and PISA are the most accurate methods for the RIM regions. PIE and PISA are statistical potentials based on the difference in distance distributions between native and incorrectly docked complexes at the residue (PIE) or atomic level (PISA). By contrast, some of the sequence-based features increased in accuracy in the Rim relative to the Core region such as the change in the count of the number of hydrogen-bond donors and acceptors and the number of aromatic residues. Finally, ΔΔG within the interior and surface regions is correlated with the change in hydrophobic and polar interfacial SASA after mutation. Although the correlation is modest here (Fig 9E and 9F), this is an important result as other features performed poorly for these regions.

### A Multiscale Approach to ΔΔG Prediction Incorporating Machine Learning

The results above suggest:

1. The difference in interface profile scores between WT and mutant structures is a relatively strong predictor of experimental ΔΔG values compared to other commonly used scoring features (Fig 4). However, it is not sufficient by itself for quantitative accuracy in prediction.
2. As a predictor of ΔΔG, the accuracy of the interface profile score can be inferred from characteristics of the profile such as the number of aligned residues at the mutation site (Fig 3A).
3. The interface profile is complementary to other scoring features for different types of mutations (Figs 5 and 9).

These features motivated us to combine the interface profile score with other scoring functions which the profile score is complementary to increase the mutation residue recognition. One common approach of the automated feature combination is machine-learning techniques which use features that are weakly predicting on their own but can be combined to give an optimal prediction of ΔΔG. We first examined whether a technique can be constructed using only the information within the interface profiles. We constructed a 13 feature set by considering 3 interface profile scores using profiles made from high and low interface similarity cutoffs (Iscore = 0.19 and Iscore = 0.25) and the adaptive interface profile along with 10 additional features reflecting the quality of the high and low interface similarity profiles. These cutoff levels were selected on the basis of validation on a separate testing dataset comprised of 20% of the data not used in validating the final result.

For the high and low interface similarity profiles we calculated additional features, including

1. The average RMSD at the mutation site.
2. The average fraction of preserved contacts after alignment relative to the number of contacts in the native complex at the mutation site.
3. The total number of aligned residues at the mutation site within the profile.
4. The relative sequence entropy at the mutation site defined by the Jenson-Shannon divergence within the profile of the amino acid distribution at the mutation site from the background amino acid distribution found in proteins.
5. The Z-score of the Jensen-Shannon Entropy relative to other interface sites.

**Fig 10:**
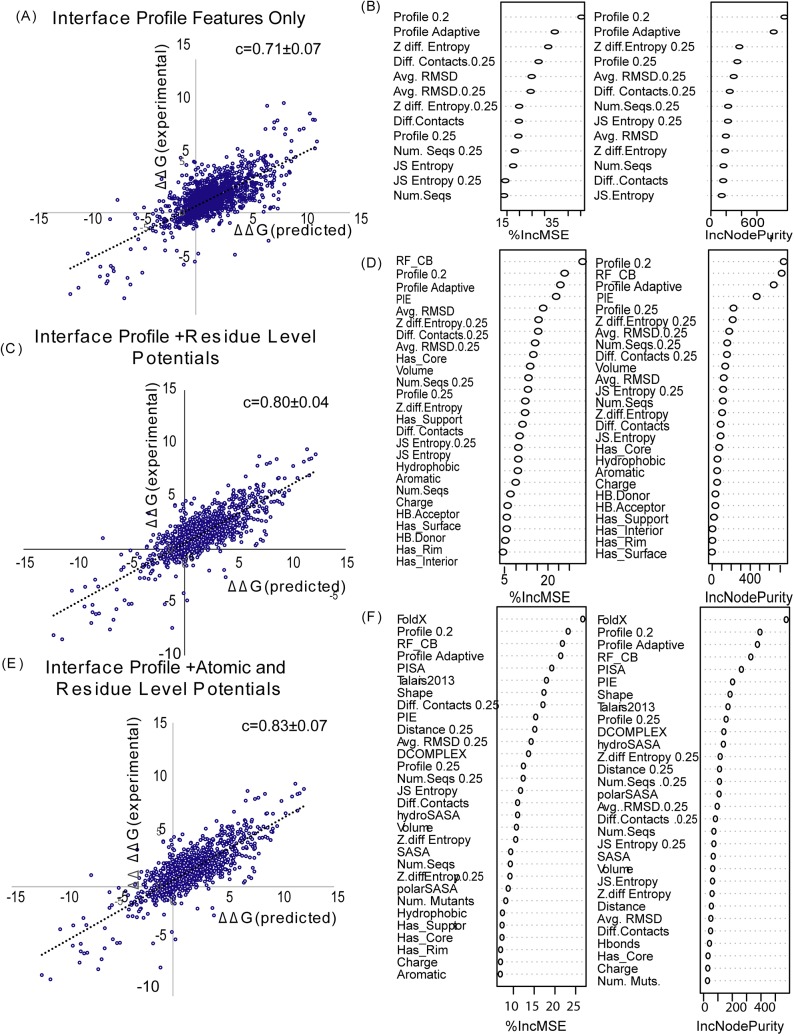
Prediction of ΔΔG value by different combinations of the interface profile scores. (A) Interface profile only; (B) Interface profile and residue level potentials; (C) Interface potential, residue level potentials, and atomic level potentials. In each picture, the right panel shows the overall correlation between predicted and experimental ΔΔG values; the right penal shows different features from random forest model as sorted by their effect on the residual error (right) or the node purity (a measure of the efficiency of splitting on feature during the construction of the decision tree) (left). Correlation values are for 10 fold cross-validation repeated three times.

The first two features report on the relative quality of the alignment of the structural profile; whether the ensemble of aligned structures actually resembles the protein complex under question or not. The last three features measure the information content within the profile and reflect whether the profile is sufficiently diverse to fully reconstruct the mutational landscape of the interaction. A random forest algorithm was then used to predict ΔΔG with these features using repeated 10 fold cross-validation (Fig 10A).

Using only the features derived from the interface profile scores, it was possible to get a correlation coefficient of *c* = 0.71±0.07 (Fig 10A) on the 10 fold cross-validated set. This level of accuracy compares favorably to the accuracy of other state-of-the-art methods [8, 14, 50, 51], despite being two orders of magnitude faster than the molecular dynamics based energy minimization methods [8, 51] and having far fewer terms than other machine learning based models [14, 50]. A true direct comparison, however, is difficult because of the different datasets used in training and different methods of cross-validation for various methods. In particular, our dataset considers both single and multiple site mutations but is only trained on dimeric complexes. A true test at the statistical significance level would require retraining each method with the specific dataset used here. Furthermore, small differences in accuracy in machine learning based methods using large amounts of features may not translate to real differences in accuracy outside of the SKEMPI dataset [16].

Nevertheless, it is possible to conclude that the structural interface profile-based method by itself can give an accuracy comparable to state of the art methods. Among the top performing methods, the Beatmusic method [9] using a combination of 13 statistical potentials weighed by solvent accessibility achieves a correlation coefficient of 0.4 on a non-redundant, single mutation set of the SKEMPI database and 0.68 after the removal of outliers. The residue level contact potential of Moal and Fernandez-Recio [14] achieves a similar performance of *c* = 0.68 when tested against the SKEMPI subset used here.

The interface profile scores and profile-based features can be incorporated with the other potentials to give an even more accurate method. We consider two additional methods, using tenfold cross-validation to confirm the results. The first method uses all the 13 profile features above and the Cβ potentials PIE and RF_CB (Fig 10B). This method has the advantage that the side-chains do not need to be calculated for each position which is the most time-consuming part of the calculation. This method has even greater accuracy than the profile only method (*c* = 0.80±0.04). Although the dominant feature in terms of determining relative error is the Cβ statistical potential RF_CB, the most important term in terms of node purity is the low interface similarity profile score and the other profile based features are also important features in the approach both in terms of relative error and node purity (Fig 10B right side). If all the terms are considered, the accuracy increases only slightly (*c* = 0.83±0.05) above the residue-level potential model (Fig 10C left side). In this model, the interface profile scores are still dominant terms (Fig 10C right side).

The standard cross-validation normally used to validate the accuracy of machine learning assumes the validation set is a non-biased subset that is representative of the actual population. In reality, the SKEMPI database is a non-representative sample of the actual protein-protein complexes. To test this bias, we performed an additional, stricter cross-validation by holding out all mutants of the proteins being tested during training [50]. This leave one out approach to cross-validation is more realistic than the standard validation process as information on mutants for the specific protein being tested is normally not available and therefore should not be included in the validation procedure. This procedure also has the effect of testing the influence of protein specific information on the model procedure and therefore serves as an indication of the overall generalizability of the model.

**Fig 11:**
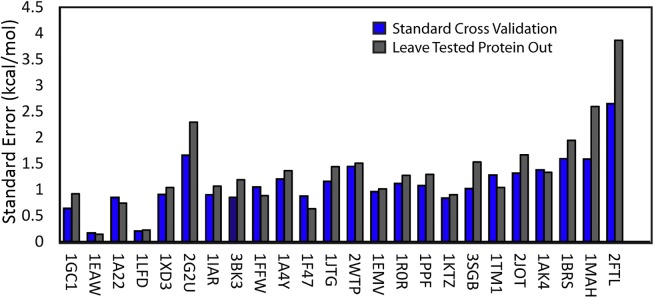
Accuracy of ΔΔG prediction on a per protein basis after leave-one-protein-out cross-validation for the 24 proteins with more than 10 mutants available based on the standard error of prediction. Proteins are arranged left to right in order from the low to high mean experimental ΔΔG value. The mean standard error across the set increases from 1.11 kcal/mol to 1.33 kcal/mol if the tested protein is left out during training.

The results of this procedure performed for the potential including all terms (Fig 10C) is shown in Fig 11 for the 24 proteins that have more than 10 mutants. The standard error of ΔΔG prediction is reported here rather than the correlation coefficient *c* as the range of ΔΔG values varies substantially among different proteins. For example, the experimental ΔΔG values for three of the proteins (1GC1, 1E22, and 1A22, left side of Fig 11) are mostly near zero (mean |ΔΔG|<0.5), indicating neutral mutations that have little effect on protein binding. The standard error of prediction is therefore more informative in this case as *c* becomes less meaningful when the values are distributed only within a narrow range.

As can be seen from Fig 11, the impact of leaving out the tested protein during training does not have a substantial impact on prediction—the mean standard error across the set increases only slightly from 1.11 kcal/mol to 1.33 kcal/mol. Such minor decrease in accuracy is smaller than the decrease seen with many other machine learning methods. For example, the accuracy of the mCSM method drops from an original cross validated standard error of 1.02 kcal/mol to 1.55 kcal/mol using a similar leave one protein out approach [50]. Overall, this accuracy is still comparable to or higher than most of the much more computationally intensive molecular dynamics based methods explicitly considering conformational flexibility [51, 58, 59].

### Limitations of the Approach

Like all mutation prediction models, the final machine-learning model has limitations. Many of the limitations are general and apply to any method that attempts to predict ΔΔG values for affinity changes by a structure-based approach. First, the model is trained only to predict ΔΔG values for dimeric complexes where mutations occur only on the side of the interface for individual complexes. While the method can be extended relatively easily to predict mutations for trimers and other types of oligomeric complexes, removing the restriction to search for linked mutations on both sides of the interface simultaneously is more difficult. Many of the terms such as the profile scores, the associated confidence measures of the profile scores, and the pharmacophore counts are strictly linearly additive with respect to the number of mutations. This assumption, which is generally not true for mutations affecting protein stability, is backed by large-scale binding selection mutagenesis experiments showing that the enrichment ratio of double mutants is strongly predicted by the enrichment ratios of the respective single mutations [60]. In these experiments, only one protein is mutated at a time corresponding to mutations on one side of the interface only. When both sides of the interface are mutated, specific interactions such as the formation of a salt-bridge across the interface can cause strong non-linearity when double mutations are compared to the sum of the respective single mutations [61]. However, for most applications one-sided mutations are of the most interest since the binding partner can be assumed to have the WT sequence since mutations are generally rare.

Finally, training and testing was performed on the SKEMPI database [16]. This database includes entries for all complexes for which a ΔΔG value and structure are available. The database does not evenly represent the universe of actual protein complexes and some protein complexes and mutation types are heavily represented while others are underrepresented. Exploring other more comprehensive datasets should help further improve BindProf.

## Discussion

Protein-protein interactions are critical for nearly every process in the cell and deleterious mutations hindering these interactions can have severe consequences for the associated cellular function. A variety of efforts from personalized medicine to understand viral evolution require knowing how specific mutations effect the protein-protein interactions. Conversely, designing proteins with improved binding or altered specificity requires that the impact of mutations on the native interface be understood. Currently this information is not available experimentally on the proteome-wide scale necessary for these tasks. Towards this end, considerable effort has been devoted towards developing methods to predict the impact of mutations on binding affinity. Most of these approaches rely on physics based methods that attempt to faithfully model on the atomic level the interactions determining protein-protein binding affinity. However, a major obstacle of such approaches is the need for the reconstruction of the full-atomic model for every mutant complex, which limits the accuracy of the approach (since the position of the side-chains is difficult to model) and reduces the computational speed and the range of applications (since rebuilding the full-atomic model is generally the most time-consuming step). In this work, we developed a novel approach, BindProf, aiming to overcome some of these limitations by introducing an interface structure profile based scoring function built on the multiple sequence alignments of analogous protein-protein interactions collected from the PDB.

Interface profile scores constructed in this manner can be used as either as a predictor of the Gibbs free energy change of protein-protein binding (ΔΔG) in their own right or combined with other features in a machine learning approach. Considered as a standalone feature, the adaptive interface profile score created by BindProf has an accuracy similar to the best all-atom potentials (Fig 4). However, unlike physics based potentials, the profile scores can be used to score thousands of mutations across a protein-protein interface very quickly (approximately 20 msec per mutation as opposed to an average of 115 seconds, for instance, for building and scoring a full atom complex by FoldX) as once the profiles are constructed the scoring of individual mutants is reduced to a very fast table lookup. In addition, the accuracy of the interface profile score can be inferred from the location of the mutation within the interface and from the characteristics of the structures used to create the profile (Fig 9). This is an advantage over current physics-based methods in which the accuracy is difficult to infer ahead of time. As such, profile scores play prominent roles in composite scoring approaches where they are combined with other features predictive of their accuracy such as the average RMSD for the aligned residues and the sequence entropy within the profile at the mutation position (Fig 10). We therefore expect that interface profiles may play important roles in future composite scoring approaches.

The effectiveness of interface profile scoring in predicting binding affinity changes has implications beyond the prediction of ΔΔG values for protein affinity changes. First, the fact that such a method can be constructed at all is independent confirmation of the results of Gao and Skolnick [62] that the existing PDB library is densely connected and approaching completeness with respect to the interface structural space, even if it is not yet complete with respect to the fold space of all possible quaternary structures. If the interface structural space of the PDB library was sparsely connected with few known structural neighbors for each complex, the profile would consist of only a few sequences and the structural profile would not be predictive of ΔΔG values. This effect can be inferred from Fig 2 when only high cutoff values are considered. Second, the degree of correlation between ΔΔG and the interface profile score bears some relationship to the degree that evolution has selected for protein binding affinity at the interface rather than other factors, although the exact relationship is obscured by the limited amount of experimental data available. As more experimental ΔΔG values are measured, profile scoring may help establish the exact role of binding affinity in evolutionary fitness. Overall, the creation of a novel evolutionary based approach with specific characteristics (including high complementarity with physics based scores, high accuracy in finding favorable mutations, low computational cost on a per mutant basis, and a relative insensitivity to side-chain conformation) should find an important application in many biomedical studies including protein design and disease-associated mutation analyses.

## Materials and Methods

### Experimental Values

Experimental ΔΔG values were derived from the SKEMPI database that consists of experimental protein affinity changes upon mutation for protein-protein complexes in which a crystal structure of the WT complex are available [16]. A subset of the database was used for testing of the interface profile scoring and multi-level machine learning. First, the selection was restricted to mutations occurring at one side of the interface to match the normal biological situation in which mutations are relatively rare and it is expected that at least one chain in the complex is WT. Since the interface profile score is fundamentally a property between two protein pairs, only dimeric complexes were selected for analysis from this set, although the method can be extended for the analysis of higher oligomeric complexes. Finally, the SKEMPI database contains multiple entries for a single mutation for 186 entries in this set. These redundant entries were averaged with outlier replicants with ΔΔG values one standard deviation above the mean disregarded. The final dataset contains 1725 entries for 130 complexes. Both single site point mutations and multiple point mutations are considered.

For random forest machine learning, three separate training, testing, and validation datasets were constructed. The training set (60% of the data) was used to construct the model, while the testing set (15% of the data) was used to tune the number of variables attempted in each split. The final model was evaluated by 10 fold cross-validation repeated three times on the validation set (25% of the data).

### Construction of FoldX Models

Crystal structures were first downloaded from the PDB and stripped of water and all non-protein ligands. A short optimization of the structure of the WT protein complex was then performed to eliminate small clashes and other undesirable features by the RepairPDB function within FoldX [43]. Structures of the mutant complex were then generated from the optimized WT structures by the BuildModel function within FoldX. The temperature for FoldX model building and energy scoring is set to the experimental temperature when known, otherwise it is set to 298 K [16].

### Calculation of Physics and Docking Based Scores

For all the sequence and physics based energies except the docking functions PIE [45], PISA [46], and DCOMPLEX [47] and the all atomic energy functions Talaris 2013 [44] and FoldX [42, 43, 63] energies were calculated separately for the mutant and WT complex structures and for both monomeric structures. The predicted ΔΔG values are then equal to:

$$\Delta \Delta G_{\text{WT}\to \text{Mut}}=[E_{WT}(\text{complex})-E_{WT}(\text{monomers})]-[E_{Mut}(\text{complex})-E_{Mut}(\text{monomers})]$$ (3)

where *E* is the relevant energy function. For the docking functions PIE, PISA, DCOMPLEX, FoldX and the Rosetta Energy function Talaris2013, this calculation is performed internally and ΔΔG is directly proportional to the difference between the energies of the two complexes:

$$\Delta \Delta G_{\text{WT}\to \text{Mut}}=E_{WT}(\text{complex})-E_{Mut}(\text{complex})$$ (4)

Changes in SASA upon mutation and number of hydrogen bonds across the interface were calculated by the Interface Analyzer in Rosetta [64].

### Construction of the Template Library

Interface structural alignment was performed using the COTH complex library of non-redundant dimeric structures. To create this library, higher order complexes in DOCKGROUND [65] are first split into all possible combinations of pairwise dimers. This is repeated for all the alternative binding modes contained within the pdb file. All dimers with either chain having less than ten interface residues are removed. The remaining structures are then filtered based on sequence and structure similarity of the complete complex to other complexes in the library. If a dimer shares at least 70% sequence identity and a TM-score at least 0.8 obtained from MM-align [66] to another structure in the complex library, it is removed from the database. The current library contains ~55000 protein-protein complexes.

### Interface Structural Similarity Metrics

Interface alignment was performed by either Ialign [33] or PCalign [34] program. The iTM-score and Iscore values are calculated by Ialign and PCscore returned by PCalign.

The equation for the interface similarity metric iTM-score is a direct analogue of the scoring matrix for TM-score [31] except that only residues within a cutoff depth of 4 Å are considered for the alignment, i.e.

$$\text{iTM-score}=\frac{1}{L_{Q}}\sum_{i=1}^{N_{a}}\frac{1}{1+d_{i}^{2}/d_{0}^{2}}$$ (5)

where *L*_*Q*_ is the total number of residues in the interface, *N*_*a*_ is the number of aligned residues, *d*_*i*_ is the distance between the Cα atoms of residues at *i*th aligned residue pair, and *d*_*0*_ is an empirical scaling factor dependent on *L*_*Q*_ to ensure the length invariance of the final score [31].

The Iscore is defined similarly except for the addition of a contact overlap factor *f*_*i*_ reflecting the fraction of conserved contacts, i.e.

$$\text{Iscore}=\frac{1}{L_{Q}}\sum_{i=1}^{N_{a}}\frac{f_{i}}{1+d_{i}^{2}/d_{0}^{2}}.$$ (6)

Here *f*_*i*_ = (*c*_*i*_/*a*_*i*_ + *c*_*i*_/*b*_*i*_)/2, where *a*_*i*_ and *b*_*i*_ are the numbers of interfacial contacts of *i*th aligned residue pair for the template and query complex, respectively, and *c*_*i*_ is the number of overlapped contacts. A contact is defined as being overlapped if the residues forming these contacts are aligned in the two pairs of chains.

The PCscore is defined analogously to the Iscore with the addition of chemical similarity measure *I*_*i*_ of *i*th residue pair:

$$\text{PCscore}=\frac{f_{c}}{L_{Q}}\sum_{i=1}^{N_{a}}\frac{1}{1+0.25(1-I_{i})+d_{i}^{2}/4^{2}}$$ (7)

where *f*_*c*_ is the ratio of common contacts between two sets of aligned interfacial residues. *I*_*i*_ equals to 1 if the *i*th pair of aligned residues are in the same chemical type, or 0 otherwise. To define the chemical equivalency, the amino acids are split into non-overlapping groups of positively charged (K, R), negatively charged (E, D), mixed hydrogen bond donor/acceptors (N, Q, S, T), aromatic (F, W), hydrophobic(C, A, I, L, M, P, V, G) and mixed donor/acceptor or aromatic (H, Y).

## Supporting Information

S1 Fig Relative frequency of ΔΔG values for different type of interface residues according to the classification in Fig 6. (PDF)
