# Supplementary material for: Predicting the Effect of Mutations on Protein-Protein Binding Interactions through Structure-Based Interface Profiles
Source: PLoS Comput Biol. 2015 Oct 27;11(10):e1004494. doi: 10.1371/journal.pcbi.1004494 (PMC4624718; doi:10.1371/journal.pcbi.1004494)
Supplement: S1 Fig — (PDF) [file pcbi.1004494.s001.pdf]

Predicting the Effect of Mutations on Protein-Protein Binding Interactions through Structure-Based Interface Profiles

Jeffrey R. Brender, Yang Zhang

Supporting Information

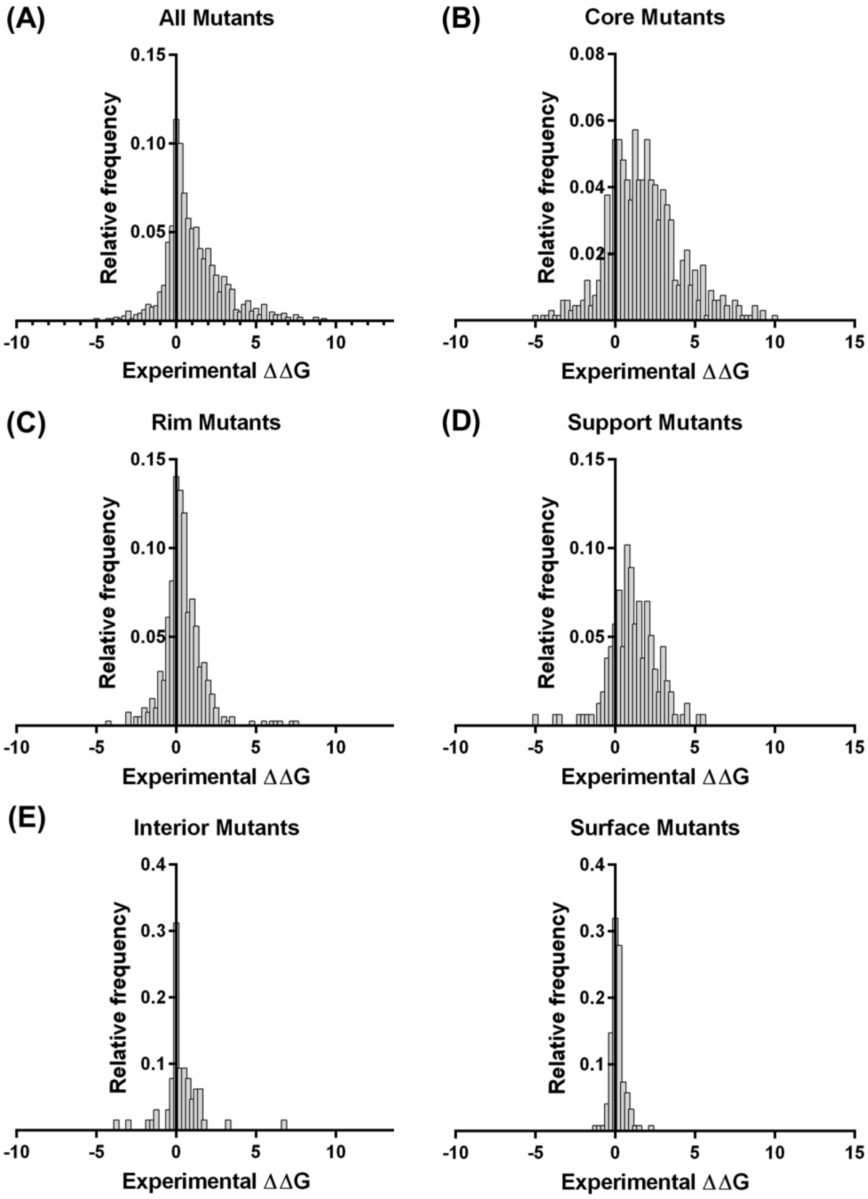

Figure S1. Relative frequency of ΔΔG values for different type of interface residues according to the classification in Figure 6.
